# Supplementary material for: Effect of Hydrothermal Aging on the Tribological Performance of Nitrile Butadiene Rubber Seals
Source: Polymers (Basel). 2023 Dec 26;16(1):81. doi: 10.3390/polym16010081 (PMC10780487; doi:10.3390/polym16010081)
Supplement: Supplementary file 1 [file polymers-16-00081-s001.zip › polymers-2769562-supplementary.pdf]

Electronic Supplementary Material (ESM)

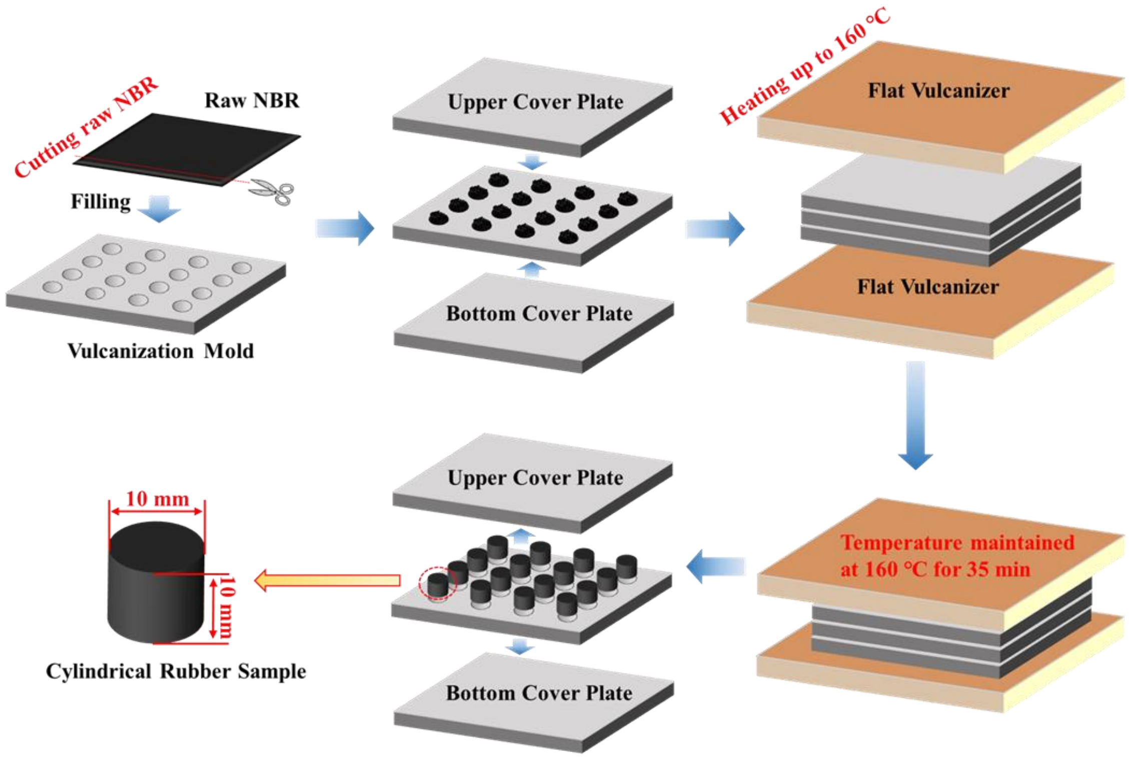

Figure S1. Schematic diagram of the NBR cylindrical samples preparation.

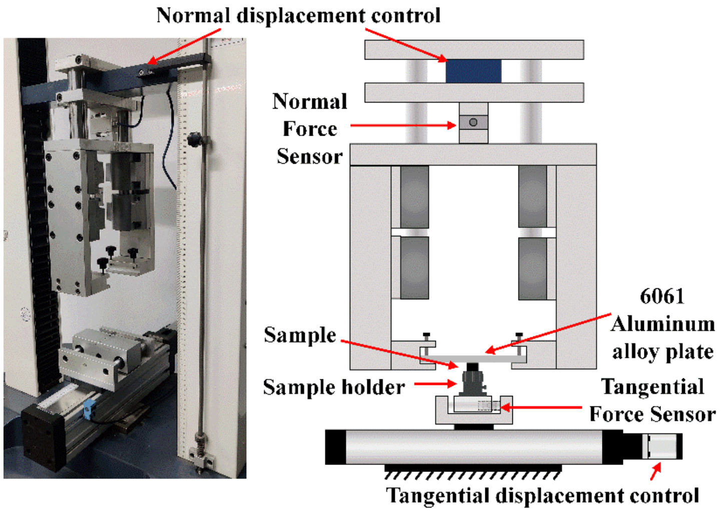

Figure S2. Schematic diagram of the modified friction testing machine.

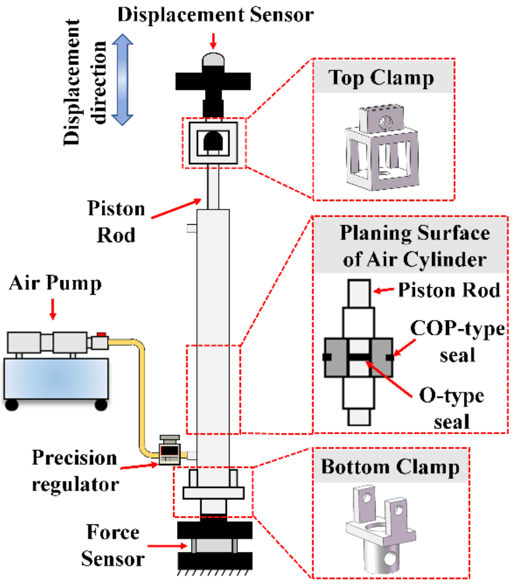

Figure S3. Schematic diagram of the cylinder dynamic friction test machine.

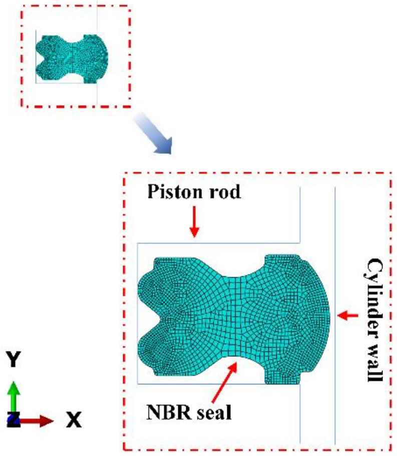

Figure S4. The simulation model of the NBR seals in the cylinder.

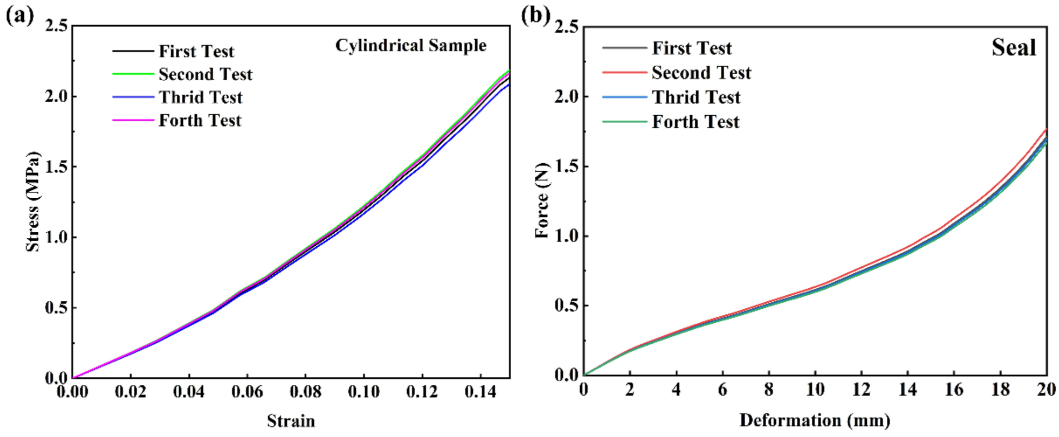

Figure S5. (a) Stress-strain curves of the NBR cylindrical samples and (b) force-deformation curves of the NBR seals with different hydrothermal aging times.

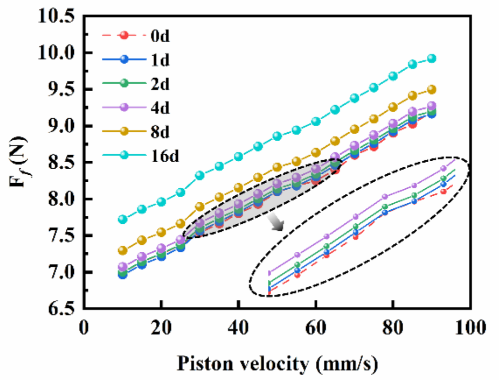

Figure S6. The F_f-piston velocity variation curves of the cylinder equipped with the aged NBR seals.

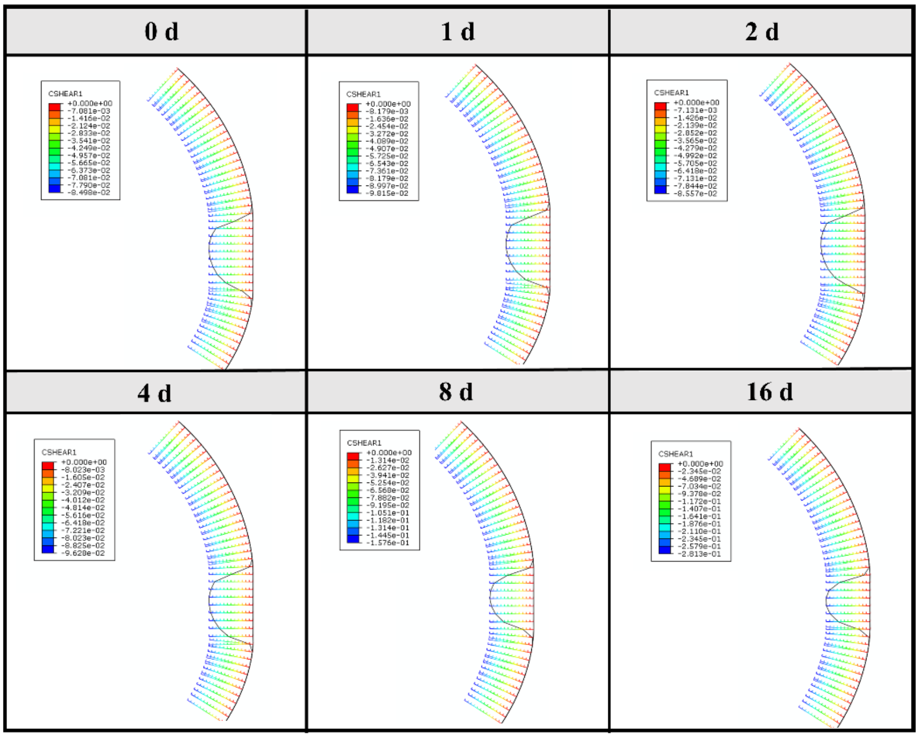

Figure S7. The shear stress distribution of the NBR seals under different hydrothermal aging times (Air pressure = 0.35 MPa).

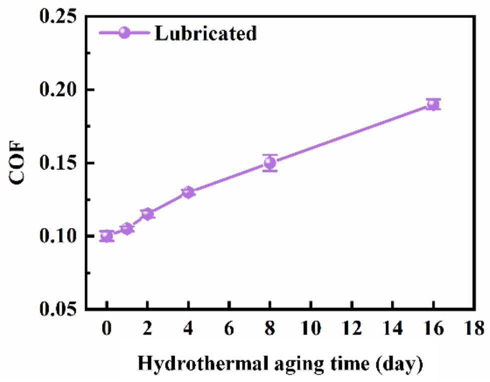

Figure S8. The variation curves of the COF of the rubber cylindrical samples with the hydrothermal aging time under lubrication conditions.

Table S1. Parameters of Mooney-Rivlin model for rubber under different hydrothermal aging times.

Aging time (day)	C_{10}	C_{01}
0	-5.22922322	5.17269996
1	2.49651156	-1.30772155
2	0.735177697	0.202485422
4	1.18789049	-1.832398799E-02
8	4.47765817	-1.95493841
16	14.3450498	-9.00759104

Table S2. The specific contact areas of the aged COP-type seals during sliding under different loading air pressures.

Air pressure (MPa)	Contact area (mm^2)				
	1 d	2 d	4 d	8 d	16 d
0.00	18.98	18.99	15.78	15.78	15.77
0.05	18.84	18.84	18.85	18.85	18.87
0.10	21.97	21.98	21.99	22.00	18.84
0.15	25.11	21.95	21.96	21.98	22.01
0.20	25.09	25.10	25.12	25.14	21.99
0.25	28.24	28.26	25.09	25.12	25.18
0.30	28.22	28.23	28.26	28.30	25.15
0.35	31.38	31.40	28.23	28.27	28.35
